# The Role of Clinicopathological Features in Tyrosine Kinase Inhibitory Duration in EGFR Mutant Metastatic Non-Small Cell Lung Cancer

**DOI:** 10.3390/jcm14041149

**Published:** 2025-02-11

**Authors:** Ender Dogan, Sedat Tarik Firat, Muhammet Cengiz, Oktay Bozkurt, Mevlude Inanc, Metin Ozkan

**Affiliations:** 1 Department of Medical Oncology, Kayseri City Education and Training Hospital, Kayseri 38080, Turkey; 2 Department of Medical Oncology, Erciyes University, Kayseri 38039, Turkey

**Keywords:** metastatic, non-small cell lung cancer, epidermal growth factor receptor, tyrosine kinase inhibitors

## Abstract

**Background:** Epidermal growth factor receptor tyrosine kinase inhibitors (EGFR-TKIs) are effective treatments for EGFR mutant (EGFRm) metastatic non-small cell lung cancer (mNSCLC). However, the benefit of EGFR-TKIs varies. We aimed to determine the impact of clinicopathological features on the duration of response to EGFR-TKIs in EGFRm mNSCLC. **Method:** Patients diagnosed with EGFRm mNSCLC who underwent EGFR-TKI therapy were retrospectively reviewed. Cox regression analyses were employed to determine the association between the PFS rates of EGFR-TKI treatments and the clinicopathological variables. **Results:** We included 83 patients in this study. The univariate analysis revealed that male gender, de novo metastatic disease, adrenal metastasis, and the absence of intrathoracic metastasis were significantly associated with poor PFS rates. The multivariate analyses revealed that male gender and adrenal metastasis were correlated with poor PFS rates. **Conclusions:** Male gender, de novo metastatic disease, adrenal metastasis, and the absence of intrathoracic metastasis negatively impact EGFR-TKI response in patients with EGFRm NSCLC.

## 1. Introduction

Lung cancer is the most commonly diagnosed cancer and the leading cause of cancer-related deaths worldwide [1]. Lung cancer is classified into two groups: small cell lung cancer and non-small cell lung cancer [2]. Non-small cell lung cancer accounts for approximately 80% of all lung cancer cases [2]. Oncogenes are important for developing non-small cell lung cancer and may be a potential therapeutic target [3]. The epidermal growth factor receptor (EGFR) pathway is a key pathway for targeted treatment. EGFR mutations cause cellular growth and differentiation by triggering the EGFR signalling pathway. EGFR-TKIs inhibit EGFR downstream signalling and trigger apoptosis [4,5]. Epidermal growth factor receptor tyrosine kinase inhibitors (EGFR-TKIs) are effective therapies that yield favourable outcomes for patients with lung cancer harbouring activating EGFR mutations [6].

Exon 19 deletion and the exon 21 L858R mutation are among the most common EGFR-TKI-sensitive mutations [7]. The first-generation (e.g., erlotinib) and second-generation (e.g., afatinib) EGFR inhibitors improve progression-free survival and objective response rate compared to those for chemotherapy [8,9]. Third-generation EGFR-TKIs (e.g., osimertinib) effectively treat EGFRm mNSCLC patients with T790M mutations. Osimertinib demonstrated improved PFS compared to that for first-generation EGFR-TKIs [10]. Some patients with EGFR-mutant non-small cell lung cancer may exhibit resistance to EGFR-TKI treatment [11], which can be primary or secondary. Pao et al. conducted a study on the reasons for EGFR-TKI resistance. They outlined several factors contributing to EGFR-TKI treatment resistance, including mutations in the EGFR exon 20 insertions, PIK3CA mutation, the loss of PTEN expression, the presence of T790M mutation, and MET amplification [11]. Additionally, the amplification of MET and HER, along with the overexpression and activation of AXL, promote resistance to EGFR-TKI treatment [12,13,14]. AXL can cause the acceleration of the emergence of T790M, and this promotes EGFR-TKI resistance [14] Another mechanism consists of its promotion of the resistance to EGFR-TKI treatment via the apolipoprotein B mRNA-editing catalytic subunit-like (APOBEC) enzyme APOBEC3A and APOBEC3B [15,16]. EGFR-TKI treatment can induce APOBEC3A and APOBEC3B expression, and this can cause EGFR-TKI resistance [15]. In a previous study, APOBEC3B/β-actin mRNA levels were found to be significantly elevated in lung cancer compared to those in normal lungs [17]. Noncoding RNAs play crucial roles in carcinogenesis and EGFR-TKI resistance by regulating the EGFR signalling pathway, including the PI3K/AKT/mTOR, Ras/Raf/MEK/ERK, JAK/STAT, and EMT pathways [18,19]. A previous study revealed that the expression of a noncoding RNA, LINC00460, is associated with a poorer response to osimertinib and serves as a poor prognostic marker for patients undergoing treatment with osimertinib [18]. These genetic alterations negatively influence EGFR-TKI response. Hung et al. conducted a study comparing survival rates between responders and nonresponders to EGFR-TKI treatment in patients with EGFRm mNSCLC [7]. They found that the nonresponders had a poor prognosis, demonstrating that bone and pleural metastases, along with a history of smoking, were unfavourable prognostic features for progression-free survival (PFS) in EGFRm mNSCLC [7]. We aimed to determine the impact of clinicopathological features on the duration of response to EGFR-TKI in patients with EGFRm mNSCLC.

## 2. Patients and Methods

### 2.1. Study Design and Patient Characteristics

Medical records of patients diagnosed with EGFRm mNSCLC who received EGFR-TKI treatment at Kayseri City Hospital and Erciyes University Department of Medical Oncology were retrospectively reviewed between January 2010 and July 2024. Patients under the age of 18, those with nonmetastatic diseases, and individuals with EGFR wild-type non-small cell lung cancer were excluded from this study. Patients with activating EGFR mutations were included in the study. EGFR mutations were detected in patients’ pathological tissue specimens. Only patients treated with erlotinib and afatinib were present in this study.

The following patient characteristics were recorded: age at the beginning of EGFR-TKI treatment, gender, ECOG performance status, type of EGFR mutation, TKI choice, TKI treatment sequence, initial metastasis status, primary tumour site, metastatic sites, and number of metastatic sites.

The patients were classified into two groups: shorter PFS and longer PFS. The shorter PFS group is characterised by PFS durations of less than 18 months in patients who received EGFR-TKI as their first-line treatment and less than 11 months in patients who received EGFR-TKI as their second-line treatment. The longer PFS group is characterised by PFS durations equal to or longer than 18 months in patients who received EGFR-TKI as their first-line treatment and equal to or longer than 11 months in patients who received EGFR-TKI in their second-line treatment. These cutoff times were determined according to median PFS durations. These groups were compared according to general characteristics.

We created a plot to illustrate the distribution of metastatic sites according to EGFR mutation types. In addition, we have included a Supplementary File that compares the general characteristics of patients with and without adrenal metastasis to determine why adrenal metastasis is a poor prognostic marker for EGFR-TKI response.

The present study was approved by the ethics committee of Kayseri City Hospital (155/30 July 2024).

### 2.2. Statistics

The descriptive statistics used for the data included the frequency and percentage for categorical variables and the median (min–max) for continuous variables. The Mann–Whitney U test was used for independent variables, and Chi-square $\left(\chi^{2}\right)$ tests were used to compare categorical variables. PFS rates for EGFR-TKI treatments were calculated using Kaplan–Meier analysis. Additionally, PFS rates based on the sequence of EGFR-TKIs and the type of EGFR mutation were performed using Kaplan–Meier analysis. Cox regression analyses were used to determine the association between the PFS rates of the EGFR-TKI treatments and other explanatory variables. PFS was defined as the time from the start of EGFR-TKI treatment until death or disease progression. Statistical significance was considered to be *p* < 0.05.

## 3. Results

We included 83 patients in the study, all of whom had non-squamous non-small cell lung cancer histology. The median age was 66 (36–87). Patients aged ≥65 comprised 53% percent of the population. In total, 51 (61%) of the patients were female, and 32 (39%) of the patients were male; 72 (87%) of the patients had an ECOG PS of 0–1, and 11 (13%) of the patients had an ECOG PS of 2. Of the patients, 75% were never smokers, 16% of the patients were ex-smokers, and 9% of the patients were smokers. The exon 19 deletion mutation was present in 60 (72%) patients, the exon 21 L858R mutation was present in 21 (25%) patients, and the exon 18 mutation was present in 3 (5%) patients. Approximately 73 (88%) of the patients were initially metastatic, and 12% of the patients were secondarily metastatic. Intrathoracic metastasis was present in 76% of the patients; liver metastasis was present in 12% of the patients; pleural metastasis was present in 40% of the patients; bone metastasis was present in 53% of the patients; adrenal metastasis was present in 17% of the patients; and brain metastasis was present 11% of the patients. In addition, lymphangitis carcinomatosis was present in 45% of the patients. The T790M mutation was present in only 37% of the patients and was the mutant in only 14% of patients after EGFR-TKI treatment progression.

In the shorter PFS group, there were 18 (47%) females and 20 (53%) males, and this group was significantly heterogeneous, with a *p*-value of 0.023. Adrenal metastasis was more prevalent in the shorter PFS group than in the longer PFS group (*p* = 0.009). Other characteristics were similar between the shorter PFS and longer PFS groups (Table 1). All of the general characteristics are shown in Table 1.

**Figure 1:**
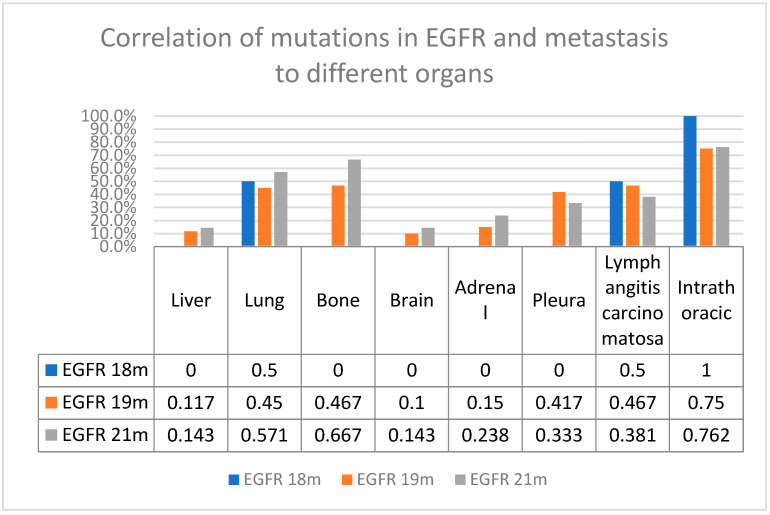
Correlation of mutations in EGFR and metastasis to different organs; m: mutation.

Liver metastasis was present in 14.3% of patients with EGFR 21m and 11.7% of patients with EGFR 19m. Lung metastasis was present in 57.1% of patients with EGFR 21m, 45% of patients with EGFR 19m, and 50% of patients with EGFR 18m. Bone metastasis was present in 66.7% of patients with EGFR 21m and 46.7% of patients with EGFR 19m. Brain metastasis was present in 14.3% of patients with EGFR 21m and 10% of patients with EGFR 19m. Adrenal metastasis was present in 23.8% of patients with EGFR 21m and 15% of patients with EGFR 19m. Intrathoracic metastasis was present in 76.2% of patients with EGFR 21m and 75% of patients with EGFR 19m. The distribution of metastatic sites according to EGFR mutation types is shown in Figure 1.

**Figure 2:**
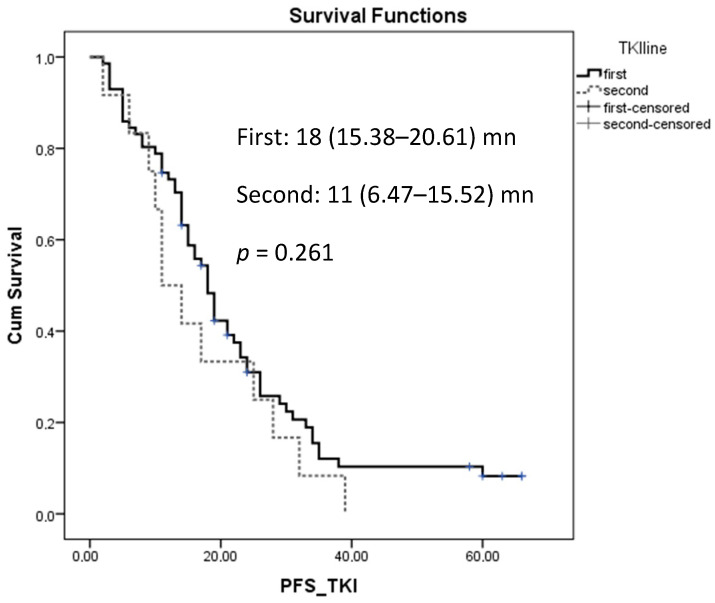
Progression-free survival of EGFR-TKIs according to treatment line.

**Figure 3:**
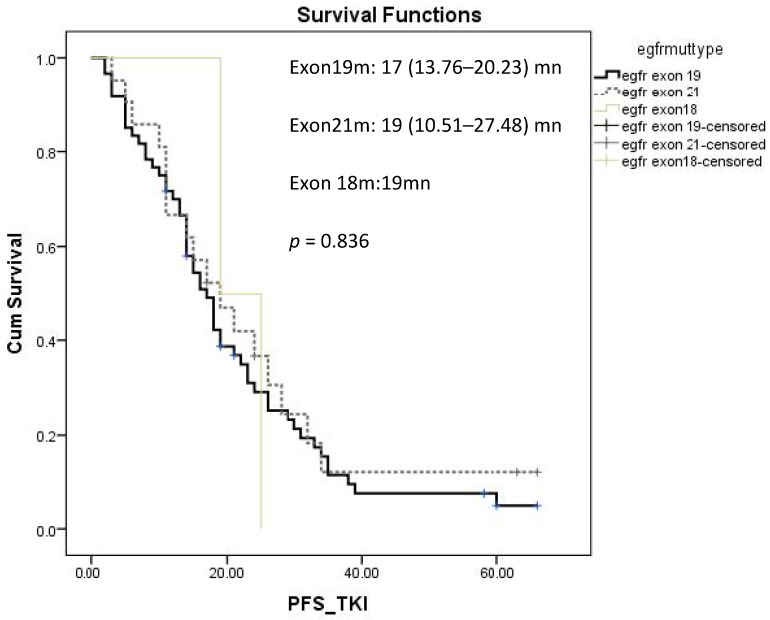
PFS of EGFR-TKIs according to mutational status.

The median PFS rate for patients who received first-line EGFR-TKI treatment was 18 (15.387–20.61) months (mn); for those who received it as a second-line treatment, this rate was 11 (6.474–15.526) mn, but it was not statistically significant (*p* = 0.261) (Figure 2). The median PFS rate of patients who received EGFR-TKI treatment in the exon 19 mutation was 17 (13.764–20.236) mn, in the exon 21 mutation, it was 19 (10.514–27.486) mn, and in the exon 18 mutation, it was 19 mn, but it was not statistically significant (*p* = 0.836) (Figure 3). Univariate analysis revealed that male gender, de novo metastatic disease, adrenal metastasis, and the absence of intrathoracic metastasis were significantly associated with poor PFS rates (Table 2). Multivariate analysis revealed that male gender and adrenal metastasis were associated with a poor PFS rate, with hazard ratios of 0.591 (0.358–0.978), *p* = 0.041, and 0.439 (0.234–0.825), *p* = 0.011, respectively (Table 2).

## 4. Discussion

Previous studies showed that EGFR-TKIs extended progression-free survival and increased objective response rates [20,21,22]. We demonstrated that the progression-free survival rate for patients who received EGFR-TKI treatment as their first-line treatment was 18 months; in the second-line treatment, it was 11 months, but this was not statistically significant. Additionally, we demonstrated that the male gender, initial metastatic disease, adrenal metastasis, and the absence of intrathoracic metastasis impacted PFS negatively in patients with EGFRm mNSCLC who received EGFR-TKI treatment in the present study.

In our study, 39% of the patients were male, and we found that male gender is one of the poor prognostic features for PFS. Hung et al. reported that male gender was associated with poor PFS in the EGFR-TKI responder group [7]. In Hung et al.’s study, the majority of patients in the EGFR-TKI responder group were female, with a percentage of 56%, while in the EGFR-TKI nonresponder group, the majority of patients were male, with a percentage of 48% [7]. There are some differences regarding drug response between males and females with NSCLC. These response differences could be explained by variations in the cancer genomics or the endogenous and exogenous factors between males and females. For example, exposure to tobacco tends to be higher in males than females, and the immune response is generally stronger in females than in males. These features may explain why a better response to EGFR-TKI is observed in females than in males [23]. Kim et al. reported a study that analysed the factors that predict the clinical benefits of EGFR-TKI therapy in patients with EGFR wild-type lung adenocarcinoma [24]. In the previous trial, osimertinib was more effective in females than in males. The hazard ratio for PFS was 0.40 (0.3–0.52) in females and 0.58 (0.41–0.82) in males [25]. In this study, the participants were mostly male, with the male gender identified as an independent poor prognostic feature. In contrast to these studies, Lin et al. did not find a statistical PFS difference between genders [26].

We found that de novo metastatic disease is an independent poor prognostic feature. In the literature, there were conflicting data about the prognostic significance of de novo metastatic disease. In contrast to our study, Dogan et al. reported that de novo metastatic disease showed no prognostic significance in EGFRm mNSCLC [27]. Ekinci et al. demonstrated that de novo metastatic disease was not a poor prognostic marker for overall survival in patients with EGFR-mutant NSCLC who received EGFR-TKI treatment [28]. Bozorgmehr et al. conducted a study that compared clinical differences between de novo and secondary metastatic EGFRm mNSCLC [29]. They found that a worse ECOG (Eastern Cooperative Oncology Group) performance status, high rates of metastatic sites, and increased rates of brain metastasis were more prevalent in de novo metastatic disease than in secondary metastatic disease [21]. Although we did not compare de novo and secondary metastatic disease based on clinicopathological features, these features could be the reason for the poor prognosis of de novo metastatic disease in our study. Additionally, Bozorgmehr found that the PFS rate was 17 months in secondary metastatic disease and 12 months in de novo metastatic disease (*p* = 0.26) [29]. Although the results did not reach a statistically significance level, secondary metastatic disease seems to have better PFS than de novo metastatic disease.

Taniguchi et al. reported that the presence of brain metastases, bone metastases, liver metastases, and pleural effusion were poor prognostic factors for patients with EGFRm mNSCLC treated with first-generation EGFR-TKIs [30]. In their study, they did not analyse adrenal metastasis. In the present study, brain, bone, liver, and pleural effusion were not statistically significant poor prognostic metastatic sites. Furthermore, adrenal metastasis was the only poor prognostic metastatic site for PFS in our study. Another study demonstrated that metastatic sites were similar between EGFR-mutant and wild-type non-small cell lung cancer types [31]. Adrenal metastasis was a poor prognostic factor for survival in patients with non-small cell lung cancer in a study by Ref. [32]. In our study, the number of metastatic sites was significantly greater in the group with adrenal metastasis than in the group without it, indicating that the tumour burden is higher in the group with adrenal metastasis than in the one without it. This could be a possible explanation for why adrenal metastasis was a poor prognostic marker. In addition, although there was no significant difference between the adrenal metastatic group and the group without adrenal metastasis with regard to brain metastasis and male gender, both brain metastasis and male gender were more prevalent in the adrenal metastatic group compared to the group without adrenal metastasis. In the present study, we also found that the absence of intrathoracic metastasis is an unfavourable feature in patients. The CEETAC study showed that extrathoracic metastasis demonstrated reduced PFS rates in patients receiving erlotinib treatment for NSCLC [33].

A previous study revealed that the EGFR-TKI treatment sequence did not influence OS, but there were different PFS rates among patients who received different lines of EGFR-TKI treatment. Utilising subgroup analysis, the study identified a longer PFS rate in patients receiving first-line EGFR-TKI treatment compared to those undergoing second-line or higher EGFR-TKI treatment among patients with advanced NSCLC with the exon 19 deletion mutation. However, this was not consistent in patients with an L858R mutation [25]. In their study, 70% of patients received second-line EGFR-TKI treatment, but only 14% of the patients received second-line EGFR-TKI treatment in our study [25]. In the present study, although the PFS rates appeared to be better in the first-line compared to the second-line treatments, the EGFR-TKI treatment sequence was not a significant unfavourable marker.

A previous study revealed that the type of EGFR mutation is an important predictor marker for PFS in patients receiving EGFR-TKI treatment. However, another study did not find a statistically significant difference between the exon 19 and 21 mutations [25,32]. Another study demonstrated that patients with the exon 19 deletion mutation had a longer PFS rate than those with the L858R exon 21 mutation [24]. In our study, mutation type was not a predictor of PFS for EGFR-TKI response.

Our study has some limitations. Firstly, the study was retrospective, and secondly, only a small patient population sample was analysed. Other limitations inlculde the absence of patients who received osimertinib treatment and the lack of knowledge about other EGFR-TKI-resistant genetic mutations (e.g., T790M).

In conclusion, in our univariate analysis, the male gender, de novo metastatic disease, adrenal metastasis, and the absence of intrathoracic metastasis were significantly associated with poor PFS rates. Multivariate analysis revealed that male gender and adrenal metastasis were correlated with poor PFS rates. These findings suggest that the characteristics of both the patients and the disease, including the coexistence of such metastases, should be considered when treating patients with EGFRm mNSCLC.

## Figures and Tables

**Table 1:** General Characteristics.

Characteristics	N = 83 (%)	Shorter PFS PFS	Longer PFS	*p*
**Age (years median min–max)**	66 (36–87)	68.5 (36–87)	64 (38–86)	0.590
**Age < 65**	39 (47)	16 (42)	23 (51)	0.509
**Age ≥ 65**	44 (53)	22 (58)	22 (49)	
**Gender**				
Female	51 (61)	18 (47)	33 (73)	0.023 *
Male	32 (39)	20 (53)	12 (27)	
**ECOG PS**				
0–1	72 (87)	32 (84)	40 (89)	0.747
2	11 (13)	6 (16)	5 (11)	
**Smoking**				
Never	62 (75)	25 (66)	37 (82)	
Ex-smoker	13 (16)	8 (21)	5 (11)	
Smoker	8 (9)	5 (13)	3 (7)	
**TKI line**				
First	71 (86)	34 (90)	37 (82)	0.533
Second	12 (14)	4 (10)	8 (18)	
**Mutation Status**				
Exon 19 del	60 (72)	30 (79)	30 (67)	0.448
Exon 21 L858R	21 (25)	8 (21)	13 (29)	
Exon 18 mutation	2 (3)	0	2 (4)	
**De novo metastatic**	73 (88)	6 (16)	3 (7)	0.289
**Metastatic site**				
Liver	10 (12)	5 (13)	5 (11)	1
Lung	40 (48)	15 (40)	25 (56)	0.187
Intrathoracic metastasis	63 (76)	27 (71)	36 (80)	0.441
Pleura	33 (40)	17 (48)	16 (36)	0.500
Bone	44 (53)	24 (63)	20 (44)	0.123
Adrenal	14 (17)	11 (29)	3 (7)	0.009 *
Brain	9 (11)	5 (13)	4 (9)	0.726
Lymphangitis carcinomatosis	37 (45)	16 (42)	21 (47)	0.825
**Number of metastatic sites**				
1	38 (46)	14 (37)	24 (63)	0.185
2 or more	45 (54)	24 (53)	21 (47)	
**Site of tumour**				
Central	38 (46)	19 (50)	19 (42)	0.514
Peripheral	45 (54)	19 (50)	26 (58)	
**T790M mutation**				
Yes	12 (14)	7 (18)	5 (11)	
No	19 (23)	10 (26)	9 (20)	
Unknown	52 (63)	21 (56)	31 (69)	
**TKI choice**				
Erlotinib	55 (66)	28 (73)	27 (60)	0.246
Afatinib	28 (34)	10 (26)	18 (40)	

* Statistical significance was considered to be *p <* 0.05.

**Table 2:** Univariate and multivariate analyses for progression-free survival on TKI.

Variables	Univariate		Multivariate	
	HR, 95% CI	*p*	HR, 95% CI	*p*
Age	0.983 (0.959–1.008)	0.188		
Age <65 vs. ≥65	1.450 (0.899–2.339)	0.127		
Gender Male or Female	0.592 (0.368–0.954)	0.031 *	0.591 (0.358–0.978)	0.041 *
ECOG PS 0–1 vs. 2	1.387 (0.686–2.805)	0.363		
Smoking Never vs. smoking history	0.845 (0.493–1.448)	0.540		
TKI treatment line First vs. second	1.415 (0.759–2.636)	0.274		
Mutation status Exon 19 vs. other	1.164 (0.689–1.969)	0.570		
De novo metastatic Yes vs. No	0.456 (0.213–0.976)	0.043 *	0.692 (0.272–1.760)	0.440
Number of metastases 1 or ≥2	1.453 (0.905–2.331)	0.122		
Liver metastasis Yes vs. No	0.662 (0.336–1.303)	0.232		
Pleura metastasis Yes vs. No	0.900 (0.559–1.452)	0.667		
Bone metastasis Yes vs. No	0.735 (0.461–1.171)	0.195		
Adrenal metastasis Yes vs. No	0.427 (0.234–0.778)	0.005 *	0.439 (0.234–0.825)	0.011 *
Brain metastasis Yes vs. No	0.693 (0.331–1.454)	0.332		
Lymphangitis carcinomatoses Yes vs. No	1.275 (0.795–2.043)	0.313		
Intrathoracic metastasis Absence vs. presence	1.94 (1.117–3.371)	0.019 *	1.535 (0.788–2.991)	0.208
Primary tumour site Central vs. peripheral	0.823 (0.518–1.309)	0.411		
TKI choice Erlotinib vs. afatinib	0.729 (0.443–1.201)	0.215		

* Statistical significance was considered to be *p* < 0.05.

## Data Availability

The raw data supporting the conclusions of this article will be made available by the corresponding author, E.D., upon reasonable request.

## Supplementary Materials

The following supporting information can be downloaded at: https://www.mdpi.com/article/10.3390/jcm14041149/s1, Table S1: General characteristics based on adrenal metastasis.

## Abbreviations

Epidermal growth factor receptor tyrosine kinase inhibitors (EGFR-TKIs); EGFR mutant (EGFRm); metastatic non-small cell lung cancer (mNSCLC); progression-free survival (PFS); ECOG (Eastern Cooperative Oncology Group); month (mn).
