# Supplementary material for: The Role of Clinicopathological Features in Tyrosine Kinase Inhibitory Duration in EGFR Mutant Metastatic Non-Small Cell Lung Cancer
Source: J Clin Med. 2025 Feb 11;14(4):1149. doi: 10.3390/jcm14041149 (PMC11857043; doi:10.3390/jcm14041149)
Supplement: Supplementary file 1 [file jcm-14-01149-s001.zip › jcm-3457818-supplementary.pdf]

Table S1: General characteristics according to status of adrenal metastasis

Characteristics	N =83 (%)	Adrenal metastasis		
		Present	Absent	*p*
**Age (years median min-max)**	66 (36-87)	68.5 (52-77)	65 (36-87)	
**Age<65**	39 (47)	6 (43)	33 (48)	0.777
**Age≥65**	44 (53)	8 (57)	36 (52)	
**Gender**				
Female	51 (61)	6 (43)	45 (65)	0.140
Male	32 (39)	8 (57)	24 (35)	
**ECOG PS**				
0-1	72 (87)	11 (79)	61 (88)	0.386
2	11 (13)	3 (21)	8 (12)	
**Smoking**				
Never	62 (75)	9 (64)	53 (77)	
Ex smoker	13 (16)	2 (14)	11 (16)	
Smoker	8 (9)	3 (22)	5 (7)	
**TKI line**				
First	71 (86)	12 (86)	59 (86)	1
Second	12 (14)	2 (14)	10 (14)	
**Mutation Status**				
Exon 19 del	60 (72)	9 (64)	51 (74)	
Exon 21 L858R	21 (25)	5 (36)	16 (23)	
Exon 18 mutation	2 (3)	0	2 (3)	
**De novo metastatic**	73 (88)	1 (7)	8 (12)	1
**Metastatic site**				
Liver	10 (12)	3 (21)	7 (10)	0.361
Lung	40 (48)	6 (43)	34 (49)	0.773
Intrathoracic metastasis	63 (76)	10 (71)	53 (77)	0.735
Pleura	33 (40)	4 (29)	29 (42)	0.389
Bone	44 (53)	9 (64)	35 (51)	0.394
Brain	9 (11)	3 (21)	6 (9)	0.173
Lymphangitis carcinomatosa	37 (45)	8 (57)	29 (42)	0.381
**Number of metastatic site**				
1	38 (46)	1 (7)	37 (54)	***0.002***
2 or more	45 (54)	13 (93)	32 (46)	
**Site of tumor**				
Central	38 (46)	6 (43)	32 (46)	1
Peripheral	45 (54)	8 (57)	37 (54)	
**T790M mutation**				
Yes	12 (14)	0	12 (17)	
No	19 (23)	5 (36)	14 (20)	
Unknown	52 (63)	9 (64)	43 (63)	
**TKI choice**				
Erlotinib	55 (66)	11 (79)	44 (64)	0.364
Afatinib	28 (34)	3 (21)	25 (36)	
